# Fascia Iliaca Block Successfully Prolonged With Dexmedetomidine and Dexamethasone for Pain Control in a Patient Undergoing Total Hip Arthroplasty

**DOI:** 10.7759/cureus.10897

**Published:** 2020-10-11

**Authors:** Nazir A Noor, Ivan Urits, Omar Viswanath, Alan D Kaye, Jonathan Eskander

**Affiliations:** 1 Anesthesiology and Critical Care, Mount Sinai Medical Center, Miami Beach, USA; 2 Anesthesia, Critical Care and Pain Medicine, Beth Israel Deaconess Medical Center, Harvard Medical School, Boston, USA; 3 Anesthesiology, Louisiana State University Shreveport, Shreveport, USA; 4 Pain Management, Valley Pain Consultants, Phoenix, USA; 5 Anesthesiology, University of Arizona College of Medicine, Phoenix, USA; 6 Anesthesiology, Creighton University School of Medicine, Omaha, USA; 7 Anesthesiology and Pain Medicine, Portsmouth Anesthesia Associates, Portsmouth, USA

**Keywords:** fascia iliaca block, dexmedetomidine, dexamethasone, dex-dex, total hip arthroplasty

## Abstract

Regional anesthesia has found many advocates as enhanced recovery after surgery continues to become a more popular option for procedures such as total hip arthroplasty. Among the many benefits is the better pain control with a reduction or complete elimination of the need for opioids for perioperative pain management. With aims to improve the multi-modal approach to pain management, we present a case demonstrating further improvements in the regional anesthetic technique with the addition of a dexamethasone and dexmedetomidine adjuvant to the local anesthetic injectate. Our case is that of a 65-year-old woman with a history of hypertension, hyperlipidemia, and right hip osteoarthritis undergoing a right total hip arthroplasty who received a preoperative ultrasound-guided fascia iliaca block with the adjuvants dexamethasone and dexmedetomidine added to the injectate. The surgery was uneventful. She did not require any postoperative opioid or non-opioid analgesics, denying any pain for the first three postoperative days.

## Introduction

The fascia iliaca block (FIB) is a favorable option for enhanced recovery after surgery (ERAS) in patients undergoing total hip arthroplasty. The use of dexamethasone alone as an adjuvant to local anesthetic has been shown to prolong the duration of action of the peripheral nerve block (PNB) in different regional anesthetic techniques. The addition of dexmedetomidine alone has also demonstrated similar findings. The literature, however, is very limited concerning the use of a combination of dexamethasone and dexmedetomidine (Dex-Dex) as adjuvants to PNB [1,2]. Zhang et al. provide one of the few studies to date demonstrating the efficacy of the Dex-Dex combination in PNB, which in their case was for an intercostal nerve block. They concluded that patients who received the Dex-Dex combination in the PNB experienced prolonged perioperative analgesia with no significant increase in adverse outcomes when compared to the groups only receiving PNB and no adjuvants, PNB with only dexamethasone, or PNB with only dexmedetomidine [3]. There are also a limited number of case reports in the literature supporting the claim that there exists a synergistic effect on the PNB’s duration of action that significantly supersedes the efficacy of using either one of these adjuvants alone [1,2,4]. Given the lack of literature regarding the Dex-Dex adjuvant in fascial plane blocks, we discuss a case resulting in successfully prolonged analgesia.

## Case presentation

**Figure 1:**
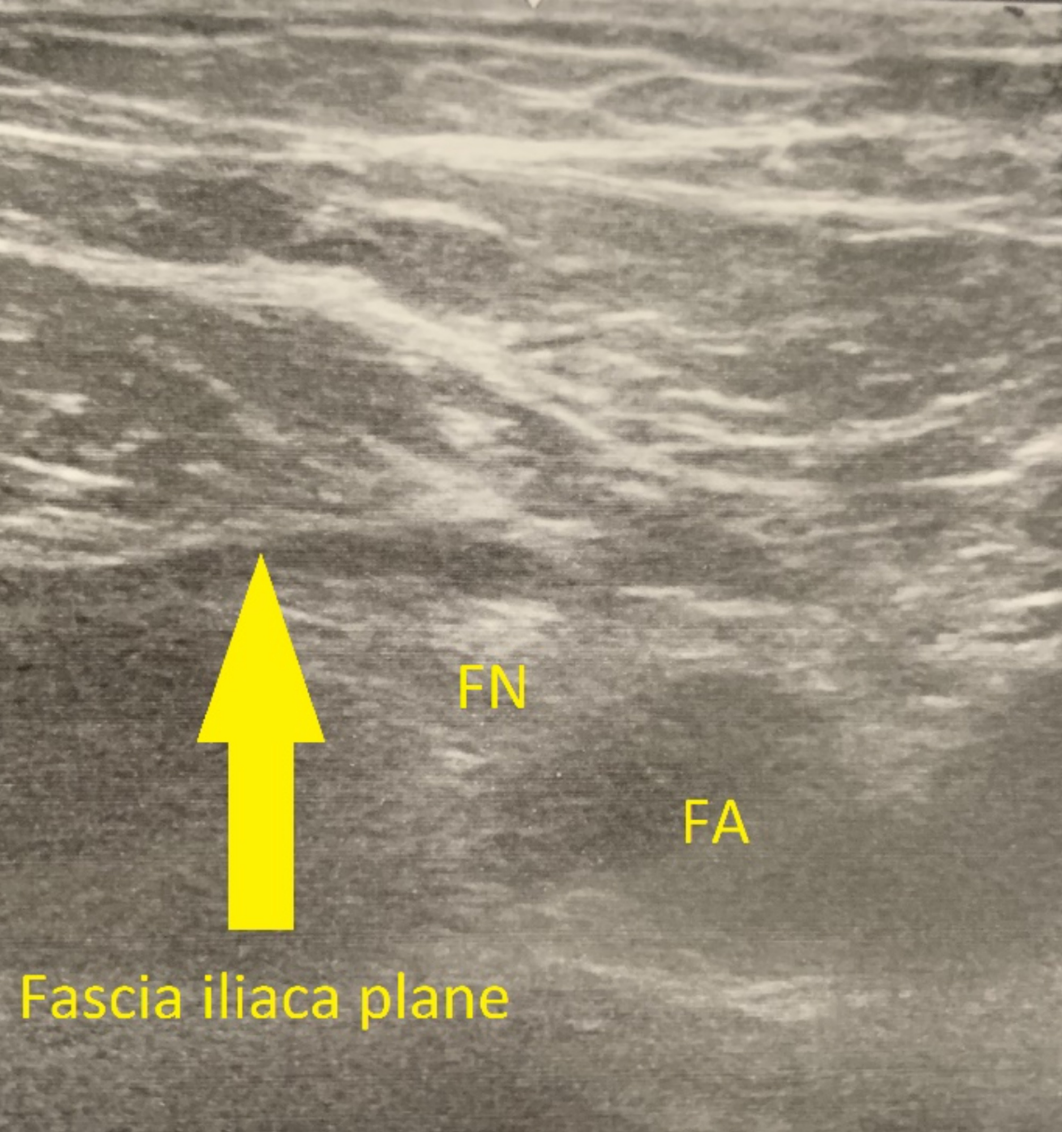
Ultrasound image of the iliacus muscle and fascia iliaca plane (yellow arrow), FN, and FA. FN, femoral nerve; FA, femoral artery

We present a case of a 65-year-old African-American woman with a history of hypertension, hyperlipidemia, and osteoarthritis of the right hip who underwent a right total hip arthroplasty. Patient consent was provided. She received a preoperative ultrasound-guided FIB on the right side (Figure 1). Once the fascia iliaca and iliacus muscle were identified through ultrasound guidance, 20 mL of 0.2% ropivacaine with 5 mg of preservative-free dexamethasone and 25 µg of dexmedetomidine was injected. The patient’s intraoperative course was uneventful. Postoperatively, she rated her pain 0/10 throughout the first 72 hours. On postoperative day 3, her pain had slightly increased from 0/10 to 2/10. The Dex-Dex adjuvant technique provided excellent opioid-sparing analgesia for our patient. It is important to note that the only opioid she received during her entire perioperative course was 100 µg of fentanyl on induction.

The mechanism of action by which dexmedetomidine provides these beneficial findings is described by Kroin et al., who postulated in her study using animal models that alpha-2-agonists cause hyperpolarization-activated cation currents which inhibit the transmission of nociceptive fibers [5]. The mechanism of action of dexamethasone in this regard is less understood, but is thought to be due to its anti-inflammatory properties [6].

## Discussion

Our case illustrates the synergistic effect of a Dex-Dex adjuvant combination with local anesthetic injectate when used in a regional fascial plane block. Zhang et al. found the analgesic effect to be prolonged with the use of the Dex-Dex technique for the intercostal nerve block [3]. Similarly improved and prolonged analgesia was demonstrated by using the Dex-Dex adjuvant for interscalene nerve blocks for surgeries of the upper extremity [4]. Our case of the total hip arthroplasty achieved similarly prolonged perioperative analgesia. The obvious difference, however, is in the type of regional block provided and the procedure that the patient underwent. Understanding that dexmedetomidine is an alpha-2-agonist, Kroin et al. describe the mechanism of action of this class of drugs as inhibiting the transmission of nociceptive fibers via hyperpolarization-activated cation currents [5]. Dexamethasone's mechanism in improved analgesia and increased blockade duration is understood to be a direct result of its anti-inflammatory properties [6].

## Conclusions

The Dex-Dex technique has demonstrated its synergistic effects as an adjuvant to PNB. However, the literature is still limited regarding its use. Our case helps advocate for the use of Dex-Dex, but further large clinical trials are necessary for a more complete understanding of the Dex-Dex technique. It has proven to be a favorable component of ERAS protocols in lower extremity procedures, such as in the total hip arthroplasty case we presented. Implementation of the Dex-Dex adjuvant has potential to provide further improvements in the duration of the analgesia, reduction or complete elimination of opioid use, and a shorter hospital course.
